# Comparison of Socioeconomic Disparities in Pump Uptake Among Children With Type 1 Diabetes in 2 Canadian Provinces With Different Payment Models

**DOI:** 10.1001/jamanetworkopen.2022.10464

**Published:** 2022-05-04

**Authors:** Jennifer M. Ladd, Atul Sharma, Elham Rahme, Kristine Kroeker, Marjolaine Dubé, Marc Simard, Céline Plante, Claudia Blais, Marni Brownell, Celia Rodd, Meranda Nakhla

**Affiliations:** 1 The Research Institute of the McGill University Health Centre, Montreal, Québec, Canada; 2 Department of Pediatrics, McGill University, Montreal, Québec, Canada; 3 Department of Pediatrics and Child Health, University of Manitoba, Winnipeg, Manitoba, Canada; 4 Children’s Hospital Research Institute of Manitoba, University of Manitoba, Winnipeg, Manitoba, Canada; 5 Department of Medicine, McGill University, Montreal, Québec, Canada; 6 Institut National de Santé Publique du Québec, Québec City, Québec, Canada; 7 Faculty of Medicine, Laval University, Québec City, Québec, Canada; 8 Faculty of Pharmacy, Laval University, Québec City, Québec, Canada; 9 Department of Community Health Sciences, University of Manitoba, Winnipeg, Manitoba, Canada; 10 Manitoba Centre for Health Policy, University of Manitoba, Winnipeg, Manitoba, Canada

## Abstract

**Importance:**

Insulin pumps improve glycemic control and quality of life in children with type 1 diabetes (T1D). Canada’s provinces have implemented universal pediatric programs to improve access. However, these programs provide differing financial coverage, allowing for unique cross-jurisdictional comparisons.

**Objective:**

To evaluate possible socioeconomic status (SES) disparities in pump uptake in Québec, where pumps are fully funded, with those in Manitoba, where pumps are partially funded.

**Design, Setting, and Participants:**

Using health administrative databases and a clinical registry, parallel, population-based cohort studies of children with diabetes were conducted from April 1, 2011, in Québec, and April 1, 2012, in Manitoba, until March 31, 2017. In analysis conducted from July 1, 2019, to November 30, 2021, multivariable Cox proportional hazards regression models were applied to study the association between pump uptake and SES, defined using validated area-based material and social deprivation indices. Children aged 1 to 17 years with T1D were identified using a validated definition in administrative data (Québec) and a clinical registry (Manitoba). Those using pumps before the initiation of provincial programs were excluded.

**Exposures:**

Socioeconomic status.

**Main Outcomes and Measures:**

Insulin pump uptake.

**Results:**

A total of 2919 children with T1D were identified in Québec: 1550 male (53.1%), mean (SD) age at diagnosis, 8.3 (4.4) years, and 1067 (36.6%) were using a pump. In Manitoba, 636 children were identified: 364 male (57.2%), mean (SD) age at diagnosis, 8.8 (4.4) years, and 106 (16.7%) were using a pump. In Québec, the mean age at diagnosis of T1D was lower in children using the pump compared with those not using a pump (7.6 [4.1] vs 8.7 [4.5] years); sex distribution was similar (562 [52.7%] vs 988 [53.3%] male). No differences in mean (SD) age at diagnosis (8.8 [4.4] vs 8.8 [4.3] years) or sex (57 [53.8%] vs 307 [57.9%] male) were noted in both groups in Manitoba. Increasing material deprivation was associated with decreased pump uptake in both Québec (adjusted hazard ratio [aHR] 0.89; 95% CI, 0.85-0.93) and Manitoba (aHR, 0.70; 95% CI, 0.60-0.82). Inclusion of ethnic concentration did not change this association. Socioeconomic disparities in pump uptake were greater in Manitoba than Québec (*P* = .006 by *t* test; Cochran *Q*, 8.15; *P* = .004; *I*^2^ = 87.7%; 95% CI, 52.5%-96.8%).

**Conclusions and Relevance:**

The results of this study suggest that the program of full coverage for pumps available in Québec partially mitigates observed SES disparities in uptake and may be a model to improve access for all children with T1D.

## Key Points

**Question:**

Are socioeconomic status disparities in insulin pump uptake in children with type 1 diabetes (T1D) different under the fully funded government program in Québec and the partially funded program in Manitoba?

**Findings:**

In parallel, population-based cohort studies including 3555 children with T1D, increasing material deprivation was significantly associated with decreased pump uptake in both Québec and Manitoba. Socioeconomic status disparities were statistically significantly greater in Manitoba than Québec.

**Meaning:**

The findings of this study suggest that full financial coverage for insulin pumps may partially mitigate the observed socioeconomic status disparities in uptake and may be a model to improve access for children with T1D.

## Teaser

This cohort study compares use of insulin pumps in children with type 1 diabetes in Québec, Canada, where full funding is available, with use in Manitoba, Canada, where funding is partial.

## Introduction

Type 1 diabetes (T1D) is a common chronic childhood disorder with significant morbidity.^1^ Continuous insulin infusion by pump has become routine care for children with T1D.^2^ Landmark studies, such as the Diabetes Control and Complications Trial/Epidemiology of Diabetes Investigations and Complications, demonstrated that intensive insulin therapy with either multiple daily injections or pumps improved glycemic control and delayed the development of complications.^3^ Despite complex technology requiring substantial self-management by the users, pumps are safe, enhance quality of life, and improve glycemic control in children with T1D.^2,4,5,6,7,8,9^ All Canadian provinces have implemented universal programs providing varying amounts of assistance to cover the cost of the pump (approximately CAD$7000) and supplies (eg, infusion sets, approximately CAD$4000 annually) for children with T1D younger than 18 years. To our knowledge, the pump program in Ontario is the only program that has been systematically evaluated.^10^ This program covers the cost of the pump and provides an annual stipend that partially covers supplies. Among Ontario children with T1D, those of lower socioeconomic status (SES) were less likely to start pump therapy and more likely to discontinue it than those of higher SES.^10^ Similar SES disparities in pump use have been seen in other nations with both universal and nonuniversal financial coverage,^4,11,12,13^ but, to our knowledge, comparisons of differing funding structures within a single country have not been performed.

The pump programs were initiated on April 1, 2011, in Québec and April 1, 2012, in Manitoba. Québec, the second largest province in Canada with a population of approximately 8.6 million,^14^ has a funding program that is unique in Canada in that it covers all pump-related costs. As in Ontario, Manitoba’s program pays for only a portion of the needed supplies, with an income-based deductible before government coverage^15^ (eTable 1 in the Supplement). Manitoba is the fifth largest province with a population of approximately 1.4 million.^14^ The distinct health care services among the provinces represents a unique opportunity for interprovincial comparisons. We hypothesized that SES disparities in pump uptake would be reduced in Québec compared with Manitoba, supporting a model of full coverage and improved access to insulin pump therapy, which is a standard-of-care technology for T1D treatment.

## Methods

### Study Design

Using multiple linked, deidentified, health-administrative databases and a clinical registry, we conducted parallel, population-based, retrospective cohort studies of children in both provinces with approval of the institutional review board of the McGill University Health Centre and the Health Ethics Research Board of the University of Manitoba with waiver of informed consent owing to use of deidentified data. The study was conducted from April 1, 2011 (Québec) or April 1, 2012 (Manitoba) to March 31, 2017. Data analysis was performed from July 1, 2019, to November 30, 2021. This study followed the Strengthening the Reporting of Observational Studies in Epidemiology (STROBE) reporting guideline for cohort studies.

### Data Sources

In Québec, linked administrative data were available at the Institut national de santé publique du Québec, using the Québec Integrated Chronic Disease Surveillance System.^16^ This system includes the Registered Persons Database (demographic characteristics) and Physician Service Claims Database (remuneration), both maintained by the Régie de l’assurance maladie du Québec, and the Hospital Discharge Database maintained by the Ministère de la Santé et des Services sociaux. In Manitoba, we used the Manitoba Population Research Data Repository,^17^ which holds linkable administrative and clinical data including the health insurance registry, hospital discharge abstracts, medical services database, and the clinical registry of Diabetes Education Resources for Children and Adolescents.

### Cohort Identification and Follow-up

We identified individuals with diabetes aged 1 to 17 years. In Québec, we used a validated definition of 4 physician visits in 1 year or 1 hospitalization with a diabetes diagnosis code to identify individuals with diabetes diagnosed from January 1, 1996 (start of data availability), to March 31, 2016.^18^ The validated definition does not distinguish between T1D and type 2 diabetes; however, in Québec, approximately 95% of children with diabetes have T1D.^18,19^ In Manitoba, we identified children with a physician diagnosis of T1D directly from the Diabetes Education Resources for Children and Adolescents clinical registry^20^ from January 1, 1986 (registry start), to March 31, 2017. Type 1 diabetes was diagnosed by the treating physician based on multiple autoantibody tests and clinical characteristics.

**Figure 1.**
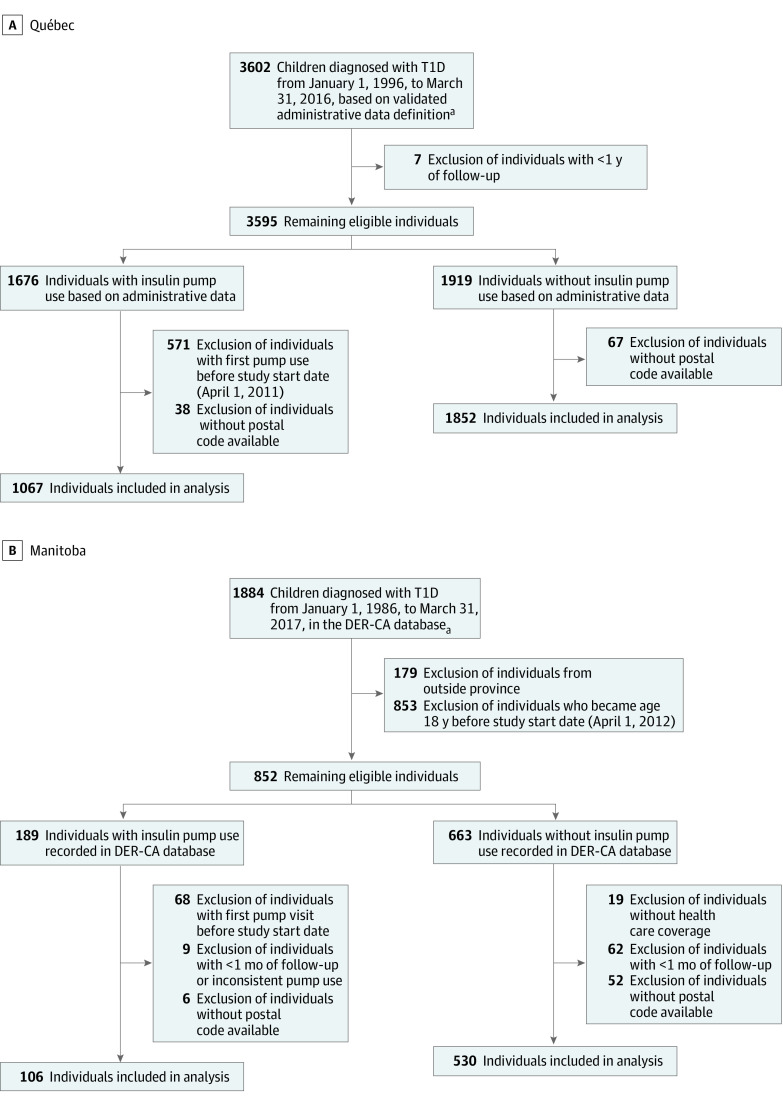
Cohort Identification Cohorts from Québec (A) and Manitoba (B). DER-CA indicates Diabetes Education Resources for Children and Adolescents; T1D, type 1 diabetes.

Individuals entered the study at either the start of the pump program (Québec: April 1, 2011; Manitoba: April 1, 2012) or the date of diabetes diagnosis, whichever was later. Follow-up ended at pump uptake, age 18 years, departure from the province, death, or the end of the study (March 31, 2017). We excluded individuals with less than 1 year of follow-up in Québec to identify those with diabetes from administrative data or less than 1 month of follow-up in Manitoba to identify those with T1D from the clinical registry. Exclusions included children with pump use before the start of the programs or those without postal codes (Figure 1; eTable 2 and eTable 3 in the Supplement).

### Primary Outcome and Exposure

Our primary outcome was pump uptake, identified by first claims of at least 2 physician pump-specific billing codes (Québec) or from the clinical registry (Manitoba). Our primary exposure was SES, determined by validated area-based deprivation indices in which individuals were assigned a material and social deprivation quintile based on their postal code at study entry, with indices uncorrelated by design.^21^ The material index considers educational level, employment status, and average income; the social index includes the proportion living alone, separated, divorced, widowed, or single parents. Material deprivation describes access to health-related goods and services, whereas social deprivation reflects social capital.^21,22^ Both material and social deprivation indices were assigned at study entry and used as continuous variables based on better model fits by Akaike information criteria.

### Covariables

Covariables included age at study entry (continuous), sex, rurality, and diagnosis era. We divided rurality into urban (population ≥10 000) or rural (<10 000) based on postal codes at study entry. Diagnosis era was based on the date of diagnosis and was divided into preprogram (before pump program implementation), early (≤24 months), or late (>24 months after implementation). Diagnosis era was included because this factor may be associated with the decision to start newer technologies, particularly if individuals received the T1D diagnosis during later eras when pumps were increasingly used. Secondary analysis included using the CAN-Marg ethnic concentration index,^23,24^ which identifies the proportion of individuals with racial and ethnic minority group status, recent immigrants, individuals born outside of Canada, and linguistically isolated individuals and has been validated for pediatric outcomes.^22^

### Statistical Analysis

Owing to provincial data-sharing restrictions that precluded any pooled analyses, each cohort was analyzed separately between 2019 and 2021. In each province, comparisons were made by Pearson χ^2^ tests for counts and *t* tests for continuous variables. The unadjusted association between pump status and SES quintiles was tested by logistic regression analysis with ordinal SES coded as orthogonal polynomials. We examined the association between SES and uptake using multivariable Cox proportional hazards regression models. A priori sample-size calculations for correlated data^25^ confirmed adequate power for adjusted hazard ratios (aHRs) less than or equal to 0.8 (Manitoba) and less than or equal to 0.93 (Québec) (eTable 4 in the Supplement). Routine diagnostic methods included tests of the proportional hazards assumption (Schoenfeld residuals) and variance inflation factors for assessing collinearity. The final model included all a priori covariates. We calculated E values for all significant results in the main analysis to assess robustness to unmeasured confounding (E values shown herein for primary exposure).^26^ In Québec, we used Kaplan-Meier curves to estimate the cumulative incidence of uptake by diagnosis era and material SES quintile. Because the curve for quintile 3 followed those of quintiles 1 and 2, SES quintiles were grouped as 1 to 3 (less deprived) and 4 to 5 (more deprived). Pairwise comparisons were made using the log-rank test with Sidak correction for multiplicity. Kaplan-Meier curves were not available for Manitoba owing to insufficient numbers.

To compare SES disparities and diagnosis era between provinces, we performed 2-sided *t* tests on their hazard estimates^27^ and applied meta-analytic methods to assess heterogeneity. The latter are designed for pooling dissimilar studies even when numbers are small.^28,29,30^ To minimize confounding from treatment inertia (eg, those with T1D diagnosed before the programs might have been reluctant to change regimens from injections to pump), we conducted a sensitivity analysis in each province limiting the cohorts to children diagnosed during the time of the programs. In addition, pump eligibility in Manitoba, but not Québec, requires a hemoglobin A_1c_ level less than 10% and no more than 1 episode of diabetic ketoacidosis (DKA) in the 12 months before pump initiation (eTable 5 in the Supplement). To assess glycemic control, we conducted a sensitivity analysis requiring a 12-month period without a DKA hospitalization before study entry. Because laboratory data were unavailable in Québec, we could not conduct a sensitivity analysis using hemoglobin A_1c_ levels. We defined DKA hospitalizations by specific *International Classification of Diseases, Ninth Revision* and *International Statistical Classification of Diseases, 10th Revision* codes and did not include DKA episodes at diagnosis. Statistical analyses were performed using SAS, version 9.4 (SAS Institute Inc) and R Studio, version 1.3.1073 (R Foundation for Statistical Analysis) using a significance threshold of *P* < .05.

## Results

### Québec

**Figure 2.**
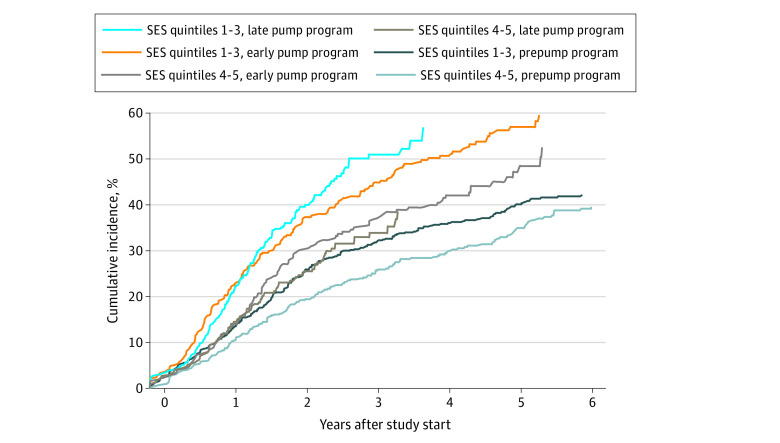
Cumulative Incidence of Pump Uptake by Material Deprivation in Québec, Stratified by Diagnosis Era *P* values for pairwise comparisons using the log-rank test with Sidak correction for multiplicity. Socioeconomic status (SES) quintiles 1 to 3 vs 4 to 5 in the prepump program diagnosis era, *P* = .99; in the early era, *P* = .06; and in the late era, *P* < .001.

We identified 2919 individuals with diabetes; mean (SD) age at diagnosis was 8.3 (4.4) years, 1550 were male (53.1%), 1369 were female (46.9%), and 1067 (36.6%) were using a pump (Table 1). Mean (SD) age at diagnosis was younger in those who obtained pumps (7.6 [4.1] years) compared with those who did not (8.7 [4.5] years); sex distribution was similar (562 [52.7%] vs 988 [53.3%] male). The unadjusted analysis showed an inverse linear association between pump uptake and increasing material (range for those using pumps: quintile 1, 237 [22.2%] vs quintile 5, 193 [18.1%]; range for those not using pumps: quintile 1, 314 [17.0%] vs quintile 5, 460 [24.8%]; *P* < .001) and social (range for those using pumps: quintile 1, 285 [26.7%] vs quintile 5, 124 [11.6%]; range for those not using pumps: quintile 1, 425 [22.9%] vs quintile 5, 318 [17.2%]; *P* < .001) deprivation quintiles. In the Cox proportional hazards regression analysis, increasing material and social deprivation were associated with decreased uptake (material deprivation: aHR, 0.89; per increase in quintile; 95% CI, 0.85-0.93, E value, 1.39; 95% CI lower limit, 1.28; social deprivation: aHR, 0.94; 95% CI, 0.90-0.98, E value, 1.26; 95% CI lower limit, 1.13) (Table 2). Diagnosis era was associated with pump uptake, with children diagnosed in the early (45%; aHR, 1.45; 95% CI, 1.24-1.68) or late (53%; aHR, 1.53; 95% CI, 1.31-1.78) era more likely to start using pumps compared with those with diabetes diagnosed before the program. Younger age at study entry was associated with lower uptake. Sex and rurality were not associated with uptake. The estimates and significance of these variables did not change with inclusion of ethnic concentration, which was significantly associated with pump uptake (Table 3). In Kaplan-Meier curves, material deprivation disparities in the cumulative incidence of users increased in the late pump era (Figure 2). In the sensitivity analysis restricted to children with diabetes diagnosed after the start of the program, increasing material deprivation was significantly associated with decreased uptake (aHR, 0.85; per quintile, 95% CI, 0.80-0.90) but social deprivation was not (aHR, 0.95; 95% CI, 0.90-1.01).

**Table 1.** Cohort Characteristics in Québec and Manitoba

Characteristic	No. (%)			*P* value^a^
	Total	Pump	No Pump	
**Québec**				
Cohort size	2919	1067 (36.6)	1852 (63.4)	
Age, mean (SD)				
At diagnosis	8.3 (4.4)	7.6 (4.1)	8.7 (4.5)	<.001
At first pump	10.9 (4.3)	10.9 (4.3)		
Sex				
Male	1550 (53.1)	562 (52.7)	988 (53.3)	.72
Female	1369 (46.9)	505 (47.3)	864 (46.7)	
Region of residence				
Rural	588 (20.1)	205 (19.2)	383 (20.7)	.34
Urban	2331 (79.9)	862 (80.8)	1469 (79.3)	
Deprivation, quintile^b^				
Material				
1	551 (18.9)	237 (22.2)	314 (17.0)	<.001
2	578 (19.8)	229 (21.5)	349 (18.8)	
3	549 (18.8)	199 (18.7)	350 (18.9)	
4	588 (20.1)	209 (19.6)	379 (20.5)	
5	653 (22.4)	193 (18.1)	460 (24.8)	
Social				
1	710 (24.3)	285 (26.7)	425 (22.9)	<.001
2	618 (21.2)	212 (19.9)	406 (21.9)	
3	605 (20.7)	245 (23.0)	360 (19.4)	
4	544 (18.6)	201 (18.8)	343 (18.5)	
5	442 (15.1)	124 (11.6)	318 (17.2)	
**Manitoba**				
Cohort size	636	106 (16.7)	530 (83.3)	
Age, mean (SD)				
At diagnosis	8.8 (4.4)	8.8 (4.4)	8.8 (4.3)	>.99
At first pump	13.4 (3.6)	13.4 (3.6)		
Sex				
Male	364 (57.2)	57 (53.8)	307 (57.9)	.45
Female	272 (42.8)	49 (46.2)	223 (42.1)	
Region of residence				
Rural	253 (39.8)	40 (37.7)	213 (40.2)	.64
Urban	383 (60.2)	66 (62.3)	317 (59.8)	
Deprivation, quintile^b^				
Material				
1	99 (15.6)	31 (29.2)	68 (12.8)	<.001
2	155 (24.4)	29 (27.4)	126 (23.8)	
3	125 (19.6)	18 (17.0)	107 (20.2)	
4	143 (22.5)	21 (19.8)	122 (23.0)	
5	114 (17.9)	7 (6.6)	107 (20.2)	
Social				
1	182 (28.6)	37 (34.9)	145 (27.4)	.04
2	107 (16.8)	23 (21.7)	84 (15.9)	
3	116 (18.2)	16 (15.1)	100 (18.9)	
4	127 (20.0)	15 (14.2)	112 (21.1)	
5	104 (16.4)	15 (14.2)	89 (16.8)	

^a^ Tests of comparison: *t* test for continuous variable of age; Pearson χ^2^ test for categorical variables of sex and regional of residence. To test for association between pump status and socioeconomic status quintiles, logistic regression was performed with ordinal SES coded as orthogonal polynomials (*P* for linear polynomial).

^b^ For each deprivation index, quintile 1 is the least deprived and quintile 5 is the most deprived.

**Table 2.** Association of Covariables With Pump Uptake in Québec and Manitoba

Variable	Adjusted HR (95% CI)^a^	*P* value
**Québec**		
Age at study entry	0.95 (0.94-0.96)	<.001
Deprivation		
Material	0.89 (0.85-0.93)	<.001
Social	0.94 (0.90-0.98)	.004
Male sex	1.02 (0.90-1.15)	.76
Urban residence	0.93 (0.79-1.10)	.42
Diagnosis era		
Early program vs preprogram	1.45 (1.24-1.68)	<.001
Late program vs preprogram	1.53 (1.31-1.78)	<.001
**Manitoba**		
Age at study entry	0.99 (0.94-1.04)	.67
Deprivation		
Material	0.70 (0.60-0.82)	<.001
Social	0.93 (0.81-1.07)	.31
Male sex	0.86 (0.58-1.26)	.43
Urban residence	0.90 (0.60-1.36)	.61
Diagnosis era		
Early program vs preprogram	5.97 (3.40-10.50)	<.001
Late program vs preprogram	3.25 (1.41-7.46)	.006

Abbreviation: HR, hazard ratio.

^a^ Adjusted multivariable Cox proportional hazards regression analysis model includes the following covariates: age at study entry, material deprivation, social deprivation, sex, rurality, and diagnosis era.

**Table 3.** Association of Covariables With Pump Uptake in Québec and Manitoba Including Ethnicity

Variable	Adjusted HR (95% CI)^a^	*P* value
**Québec^b^**		
Age at study entry	0.95 (0.94-0.96)	<.001
Deprivation		
Material	0.90 (0.86-0.94)	<.001
Social	0.95 (0.91-0.99)	.02
Ethnic concentration	0.90 (0.86-0.95)	<.001
Male	1.00 (0.89-1.13)	>.99
Urban residence	1.09 (0.91-1.31)	.95
Diagnosis era		
Early program vs preprogram	1.44 (1.24-1.68)	<.001
Late program vs preprogram	1.50 (1.29-1.75)	<.001
**Manitoba^c^**		
Age at study entry	0.99 (0.94-1.04)	.65
Deprivation		
Material	0.75 (0.63-0.90)	.002
Social	0.88 (0.75-1.02)	.078
Ethnic concentration	0.98 (0.71-1.35)	.89
Male sex	0.82 (0.56-1.22)	.33
Urban residence	1.04 (0.64-1.69)	.88
Diagnosis era		
Early program vs preprogram	5.97 (3.32-10.75)	<.001
Late program vs preprogram	3.69 (1.57-8.62)	.003

Abbreviation: HR, hazard ratio.

^a^ Adjusted multivariable Cox proportional hazards regression analysis model includes the following covariates: age at study entry, material and social deprivation indices as continuous variables, ethnicity (CAN-Marg ethnic concentration index as continuous variable), sex, rurality, and diagnosis era.

^b^ Twenty additional individuals with missing or unavailable CAN-Marg ethno-cultural composition index; thus, the remaining cohort included 1055 children who used a pump users and 1844 who did not use a pump.

^c^ Twenty-six additional individuals with missing or unavailable CAN-Marg ethno-cultural composition index; thus, the remaining cohort included 102 children who used a pump users and 508 who did not use a pump.

### Manitoba

We identified 636 individuals with T1D; mean (SD) age at diagnosis was 8.8 (4.4) years; 364 were male (57.2%), 272 were female (42.8%), and 106 (16.7%) were using a pump (Table 1)—significantly less than the proportion in Québec (*P* < .001). The mean (SD) age at diagnosis was the same in both the pump (8.8 [4.4] years) and no pump (8.8 [4.3] years) groups. As in Québec, the unadjusted analysis showed an inverse linear association between pump uptake and increasing material (range for those using a pump: quintile 1, 31 [29.2%] vs quintile 5, 7 [6.6%]; range for those not using a pump: quintile 1, 68 [12.8%] vs quintile 5, 107 [20.2%]; *P* < .001) and social (range for those using a pump: quintile 1, 37 [34.9%] vs quintile 5: 15 [14.2%]; range for those not using a pump: quintile 1, 145 [27.4%] vs quintile 5, 89 [16.8%]; *P = * .04) deprivation quintiles. In the Cox proportional hazards regression analysis, material deprivation was associated with decreased uptake (aHR, 0.70; per increase in quintile, 95% CI, 0.60-0.82; E value, 1.88; 95% CI lower limit, 1.56) (Table 2). As in Québec, diagnosis era was significantly associated with uptake in the early era (aHR, 5.97; (95% CI, 3.40-10.50) and the late era (aHR, 3.25; 95% CI, 1.41-7.46) compared with preprogram diagnosis. Social deprivation, age, sex, and rurality were not associated with uptake. Inclusion of ethnic concentration in the model did not alter these results, and ethnic concentration was not significantly associated with pump uptake (Table 3). Results were comparable when restricted to the cohort diagnosed during the pump program.

### Québec and Manitoba Comparison

The 2 cohorts had similar SES profiles with mean (SD) material deprivation quintile 3.08 (1.43) in Québec and 3.03 (1.34) in Manitoba (*P* = .40). The SES disparities in pump uptake differed significantly between provinces, with Manitoba demonstrating a more significant decrease in uptake with increasing material deprivation compared with Québec (*P* = .006; E value, 1.78; 95% CI lower limit, 1.32). We noted significant heterogeneity between provinces in material deprivation (Cochran *Q*, 8.15; *P* = .004; *I*^2^ = 87.7%; 95% CI, 52.5%-96.8%). The sensitivity analysis requiring a 12-month period without a DKA hospitalization before study entry demonstrated no notable differences from the main Cox proportional hazards regression analyses (eTable 6 in the Supplement), and the interprovincial comparison was unchanged. There was no significant interprovincial difference in the aHR for social deprivation. There was a statistically significant difference between provinces for the association between early program era and pump uptake (Québec aHR, 1.45; 95% CI, 1.24-1.68; and Manitoba aHR 5.97; 95% CI, 3.40-10.50: *P*<.001).

## Discussion

In this population-based cohort study, we noted significant SES disparities in insulin pump uptake in 2 Canadian provinces with similar median family incomes^31^ and comparable material deprivation quintiles, but with differing governmental funding structures for pumps and associated supplies. Increasing material deprivation was significantly associated with decreasing uptake in both Québec, where pumps and supplies are fully covered, and Manitoba, where partial coverage is provided. The association between increasing material deprivation and decreasing pump uptake was more pronounced in Manitoba, suggesting that the comprehensive financial coverage provided by Québec may mitigate observed SES disparities in uptake and may represent a model for other governments seeking to improve universal access.

Despite relatively greater uptake in Manitoba than Québec in the early program era (perhaps associated with the increase in support from nonphysician health professionals in the Manitoba program), overall uptake appears to be much lower in Manitoba than in Québec, with the latter more consistent with experiences of other provinces.^10^ This difference may be associated with barriers to travel to a centralized pump training center in Manitoba. In addition, although both governments require ongoing assessment of patient eligibility to enroll in pump programs (eg, regular glucose testing and continued follow-up), Manitoba requires at least 3 recent hemoglobin A_1c_ levels less than 10% before pump initiation. However, our findings of SES disparities did not change when the cohorts were restricted by requiring a 12-month period without DKA hospitalization before study entry, which is a more rigorous measure of control than used in practice.

Our observed SES disparities in uptake are similar to those in other countries. In New Zealand, which publicly funds pumps and supplies similarly to Québec, increased deprivation, as determined by a validated area-based measure, was associated with reduced pump use.^11,12^ In Germany, which also has full coverage, lower pump use was reported in those of lower SES as measured by individually reported income, educational level, and employment.^13^ In the US, where there is no unified coverage, numerous studies, including SEARCH for Diabetes in Youth, have shown an association between pump use and higher income and private insurance—markers of higher SES.^4,32,33,34,35^

Because worsening material deprivation is a factor associated with disparities in pump uptake despite comprehensive financial support in Québec, disparities may not be strictly owing to monetary concerns. Transportation difficulties or work obligations may prevent families from attending clinic or pump education sessions, with missed opportunities for learning and guidance. The material deprivation index also includes education, and a lower parental educational level has been associated with decreased uptake.^4,35^ A survey of US parents of children with T1D not using pumps reported that parental concerns include the perceived complexity of the technology,^35^ and another US study found language barriers were significantly associated with lower use.^36^ Furthermore, in adjusted analysis in Québec, but not in Manitoba, disparities in ethnic concentration and social deprivation were found in uptake. Similar ethnic disparities, independent of SES, have been described in the US in T1D Exchange studies^37,38^ and in New Zealand.^11,12^ Another study in the US found an association between intact family structure and increased pump use.^39^ Taken together, although comprehensive government financial support seems to decrease some disparities in uptake, additional supports are needed to improve equity by addressing barriers, such as those related to ethnic minority status and educational level.

### Limitations

This study has limitations. Although our study included available data from all children in Québec and Manitoba with universal health insurance, identification of individuals with diabetes using a pump was inherently different in the 2 provinces. However, methods of identification had been previously validated,^18,20^ and exclusion criteria were consistent in both provinces.

Because the administrative data of Québec did not include laboratory parameters, we were unable to adjust for hemoglobin A_1c_ levels. However, diabetes is an ambulatory care–sensitive condition for which quality outpatient care and optimal access to care can potentially prevent complications.^40^ We therefore used DKA as a proxy measure of glycemic control since poor control and DKA hospitalizations have overlapping causes, including suboptimal access to outpatient care or adherence to management.^40,41^ Future studies incorporating clinical data are needed to assess the association between glycemic control and pump uptake in the setting of differing funding supports.

In addition, in this large population-based study, SES was defined at a neighborhood rather than individual level, which may result in misclassification. The cohort size was smaller in Manitoba than Québec—a reflection of provincial populations—which limits power. We did not have information on family preferences and health care professional biases, which could influence uptake directly and mediate SES disparities in pump uptake. Eligible patients may choose not to start pump therapy for a variety of reasons, including reluctance to wear visible devices.^42^ Similarly, health care professionals may not offer pumps to marginalized populations or families with financial difficulties or challenges with adherence to diabetes management due to unconscious biases.^43^ Given the observational design, unmeasured confounders are possible despite E values greater than 1. Our observed SES disparities in pump uptake are likely present for other wearable devices, such as continuous glucose monitoring systems.^44^ However, the Québec administrative data did not include continuous glucose monitoring information. Future research should examine SES disparities in these devices across same-country jurisdictions and explore the contribution of patient and health care professional factors on disparities in diabetes technology use.

## Conclusions

Insulin pump therapy is a mainstay of efforts to reduce long-term morbidity and improve quality of life in individuals with T1D. Previous studies within universal financial coverage health care systems have documented SES disparities in uptake, but, to our knowledge, none have compared jurisdictions in the same country with varying degrees of coverage. We found that SES disparities in pump uptake appear to be mitigated in Québec, which has comprehensive government financial support, compared with Manitoba, which has partial coverage for supplies. This finding suggests that other provincial and national programs can improve treatment access for individuals of lower SES through more comprehensive coverage. Programs aimed at reduction of educational, ethnic, and monetary disparities are essential to improve health equity, and further research into family preferences and health care professional biases are needed to optimize the care of children with T1D.

## Notes

Supplement. **eTable 1.** Example Deductible for an Economic Family (Two Parents, One Child) in Manitoba **eTable 2.** Characteristics of Individuals Missing Postal Codes in Québec **eTable 3.** Characteristics of Individuals Missing Postal Codes in Manitoba **eTable 4.** Sample Size Calculations for Cox Analysis **eTable 5.** Highlights of Pump Program Eligibility Criteria in Québec and Manitoba **eTable 6.** Sensitivity Analysis Accounting for DKA Hospitalizations
