# Supplementary material for: Comparison of Socioeconomic Disparities in Pump Uptake Among Children With Type 1 Diabetes in 2 Canadian Provinces With Different Payment Models
Source: JAMA Netw Open. 2022 May 4;5(5):e2210464. doi: 10.1001/jamanetworkopen.2022.10464 (PMC9069256; doi:10.1001/jamanetworkopen.2022.10464)
Supplement: Supplement. — eTable 1. Example Deductible for an Economic Family (Two Parents, One Child) in Manitoba eTable 2. Characteristics of Individuals Missing Postal Codes in Québec eTable 3. Characteristics of Individuals Missing Postal Codes in Manitoba eTable 4. Sample Size Calculations for Cox Analysis eTable 5. Highlights of Pump Program Eligibility Criteria in Québec and Manitoba eTable 6. Sensitivity Analysis Accounting for DKA Hospitalizations [file jamanetwopen-e2210464-s001.pdf]

Supplementary Online Content

Ladd JM, Sharma A, Rahme E, et al. Comparison of socioeconomic disparities in pump uptake among children with type 1 diabetes in 2 Canadian provinces with different payment models. *JAMA Netw Open*. 2022;5(5):e2210464. doi:10.1001/jamanetworkopen.2022.10464

eTable 1. Example Deductible for an Economic Family (Two Parents, One Child) in Manitoba

eTable 2. Characteristics of Individuals Missing Postal Codes in Québec

eTable 3. Characteristics of Individuals Missing Postal Codes in Manitoba

eTable 4. Sample Size Calculations for Cox Analysis

eTable 5. Highlights of Pump Program Eligibility Criteria in Québec and Manitoba

eTable 6. Sensitivity Analysis Accounting for DKA Hospitalizations

This supplementary material has been provided by the authors to give readers additional information about their work.

© 2022 Ladd JM et al. *JAMA Network Open.*

eTable 1: Example deductible for an economic family (two parents, one child) in Manitoba [a]

Median Income	$84,441
Pharmacare Deductible<br>(i.e., amount family or supplemental private insurance must pay before Manitoba's pump program pays for supplies)	$5608.58

[a]Based on 2015 Statistics Canada data for median income and 2021 Manitoba Pharmacare Deductible Estimator Results

© 2022 Ladd JM et al. *JAMA Network Open.*

eTable 2: Characteristics of individuals missing postal codes in Québec

Characteristic		Individuals Excluded	Individuals Included in Cohort	*p*-value
Total, No.		105	2919	
Age at diagnosis, mean (SD)		11.2 (4.2)	10.4 (4.2)	0.07
Sex, No. (%)	Male	60 (57.1)	1550 (53.1)	0.40
	Female	45 (42.9)	1369 (46.9)	
Diagnosis Era, No. (%)	Pre-Program	58 (55.2)	1485 (50.9)	0.41
	Early Program	23 (21.9)	581 (19.9)	
	Late Program	24 (22.9)	853 (29.2)	
Pump User, No. (%)	Yes	38 (36.2)	1067 (36.6)	0.94
	No	67 (63.8)	1852 (63.4)	

Note: SD = standard deviation, No. = number, % = percentage

© 2022 Ladd JM et al. *JAMA Network Open.*

eTable 3: Characteristics of individuals missing postal codes in Manitoba

Characteristic		Individuals Excluded	Individuals Included in Cohort	*p*-value
Total, No.		58	636	
Age at diagnosis, mean (SD)		8.2 (3.9)	8.8 (4.3)	0.30
Sex, No. (%)	Male	35 (60.3)	364 (57.2)	0.65
	Female	23 (39.7)	272 (42.8)	
Duration of diabetes, mean (SD)		5.4 (4.5)	5.3 (3.9)	0.76
Pump User, No. (%)	Yes	6 (10.3)	106 (16.7)	0.20
	No	52 (89.7)	530 (83.3)	

Note: SD = standard deviation, No. = number, % = percentage

© 2022 Ladd JM et al. *JAMA Network Open.*

eTable 4: Sample size calculations for Cox analysis[a]

Québec	Manitoba
Assuming<br>• N ~ 3000 = total sample<br>• σ^2 = 1.42 = variance of the covariate of interest (material deprivation quintile). This estimated is based on previous studies conducted by the authors or by assuming uniform distribution across quintiles.<br>• ψ ~ 1000/3000 = the proportion reaching 'end-point' i.e., pump uptake<br>• R^2 = 0.25 = square of the multiple correlation coefficient between the covariate of interest and other covariates (assumed zero for a randomize trial)<br>• α=0.05=type I error<br><br>We are adequately powered to detect a hazard ratio of ≤0.93. Sensitivity analysis shows this conclusion is relatively robust to assumed values of R^2 between 0.0-0.5 (minimum effect size = HR between 0.92 - 0.94).	Assuming:<br>• N ~ 600 = total sample<br>• σ^2 = 1.42 = variance of the covariate of interest (material deprivation quintile). This estimated is based on previous studies conducted by the authors or by assuming uniform distribution across quintiles.<br>• ψ ~ 100/600 = the proportion reaching 'end-point' i.e., pump uptake<br>• R^2 = 0.25 = square of the multiple correlation coefficient between the covariate of interest and other covariates (assumed zero for a randomize trial)<br>• α=0.05=type I error<br><br>We are adequately powered to detect a hazard ratio of ≤0.8. Sensitivity analysis shows this conclusion is relatively robust to assumed values of R^2 between 0.0-0.5 (minimum effect size = HR between 0.76 - 0.82).

[a]*A priori* application of the power calculation for the Cox model with continuous covariates from Hsieh and Lavori, 2000. Principal covariate of interest is the INSPQ material deprivation quintile.

© 2022 Ladd JM et al. *JAMA Network Open.*

eTable 5: Highlights of pump program eligibility criteria in Québec and Manitoba

Québec	Manitoba
- T1D<br>- Under age 18 years<br>- Has government insurance coverage<br>- Regular monitoring of blood glucose<br>- Mastery of advanced carbohydrate counting<br>- Participation in pump education training program at diabetes center<br>- Follow-up with diabetes care team at least 3 times per year<br>- Clinical eligibility confirmed by a physician	- T1D<br>- Under age 18 years<br>- Has government insurance coverage<br>- Regular monitoring of blood glucose<br>- Mastery of advanced carbohydrate counting<br>- Participation in pump education training program at diabetes center<br>- Follow-up with diabetes care team at least 3 times per year<br>- Clinical eligibility confirmed by a physician<br>- No more than 1 episode of DKA in prior 12 months<br>- 3 most recent HbA1c < 10%

Note: T1D = type 1 diabetes, DKA = diabetic ketoacidosis, HbA1c = hemoglobin A1c

© 2022 Ladd JM et al. *JAMA Network Open.*

eTable 6: Sensitivity analysis accounting for DKA hospitalizations[a]

Variable		Québec[b] Adjusted HR[d] (95% CI)	Québec[b] *p*-value	Manitoba[c] Adjusted HR[d] (95% CI)	Manitoba[c] *p*-value
Age at Study Entry		0.95 (0.93 – 0.97)	<0.001	1.26 (1.16 – 1.37)	<0.001
Material Deprivation		0.90 (0.85 – 0.94)	<0.001	0.76 (0.65 – 0.89)	0.001
Social Deprivation		0.93 (0.88 – 0.97)	0.003	0.89 (0.77 – 1.04)	0.14
Male		0.99 (0.86 – 1.14)	0.89	0.80 (0.53 – 1.20)	0.28
Urban		0.96 (0.80 – 1.16)	0.67	1.27 (0.83 – 1.96)	0.27
Diagnosis Era	Early Program vs. Pre-Program	1.36 (1.14 – 1.63)	<0.001	3.37 (2.06 – 5.49)	<0.001
	Late Program vs. Pre-Program	1.66 (1.38 – 2.00)	<0.001	3.28 (1.18 – 9.09)	0.02

Note: HR = hazard ratio, CI = confidence interval
[a] *To ensure that children from both provinces satisfied the same eligibility criteria in terms of glycemic control, exclusion criteria and end date of follow-up were as in the main Cox analysis except that a 12 month period without DKA was required for cohort entry (excluding DKA at diagnosis)and children were censored at DKA during follow-up*
[b]*Remaining cohort included 800 pump users and 1880 non-pump users*
[c]*Remaining cohort included 96 pump users and 457 non-pump users*
[d]*Adjusted multivariable Cox regression analysis model includes the following covariates: age at study entry, material deprivation and social deprivation (INSPQ deprivation indices as continuous variables), sex, rurality, and diagnosis era.*

© 2022 Ladd JM et al. *JAMA Network Open.*
